# Valorization of Moroccan *Crocus sativus* L. By-products: Foliar Spraying of Aqueous Tepal Extract Stimulates Growth and Confers Antioxidant Properties in Eggplant Seedling under Greenhouse Conditions

**DOI:** 10.1155/2020/8812157

**Published:** 2020-11-05

**Authors:** Amine Khoulati, Sabir Ouahhoud, Samira Mamri, Mouhssine Meziane, Mohammed Choukri, Abdeslam Asehraou, Ennouamane Saalaoui

**Affiliations:** ^1^Laboratory of Biochemistry and Biotechnology, Faculty of Sciences, Mohammed First University, BP 717, Oujda 60000, Morocco; ^2^Laboratory of Biochemistry, University Hospital Center Mohammed VI, BP 4806, Oujda 60000, Morocco

## Abstract

The valorization of *Crocus sativus* L. by-products has become important given its interesting content of bioactive molecules. In the present study, aqueous tepal extract (ATE) studied eggplant seedling's growth and physiology under a plastic tunnel. ATE was foliage sprayed 3 times every 15 days, with various concentrations (1 mg/mL, 2 mg/mL, 3 mg/mL) in addition to a treatment containing 2 mg/mL of tepals and 0.6 mg/mL of stigmas (T+S). The concentration of 2 mg/L ATE significantly (*p* ≤ 0.05) increased the plant's height, the chlorophyll content, and decreased antioxidant activity and MDA (malondialdehyde). However, the concentration 3 mg/mL inhibited the plant growth; increased the content of ascorbic acid and polyphenol; and resulted in lipid peroxidation and antioxidant activities increases, indicating oxidative stress (*p* ≤ 0.05). On the other hand, T+S significantly influenced some parameters analyzed. Our findings demonstrate that ATE can act as a biostimulant at 2 mg/mL to enhance eggplant growth in plastic tunnel production and used in plant stress situations.

## 1. Introduction

Saffron (*Crocus sativus* L.), also called “red gold,” is a geophytic herbaceous plant and is one of the most expensive commercial spices belonging to the Iridaceae family [1]. Saffron grows on different soil types with pH ranges from neutral to slightly alkaline and well-drained [2]. Commonly, planting is done by hand or with the machines used for potato or onion. Planting is carried out at a depth of 10–20 cm, with a spacing of 10–20 cm between bulbs and 20–30 cm between rows. In Morocco, saffron is manually planted in rows 20 cm apart, with rows spaced 10 to 15 cm apart and planting depth of about 15 cm. During the harvest period, the closed flowers are picked by hand in the morning to obtain a spice with high qualitative traits and preserving the crocin content responsible for the color and the safranal content accountable for the odor. Many researchers have focused their attention on valuing saffron by-products to increase crop profitability, such as floral bioresidues (tepals) [3]. This perspective is receiving increasing attention in the cultivation of saffron using its by-products, specially tepals rich in bioactive molecules such as polyphenol, flavonols, and anthocyanins [4]. In the production of saffron, for each kilogram of spices produced, about 53 kg of tepals are made [5], and 90% of the total fresh weight corresponds to the by-products composed of tepals, which are generally discarded as waste [6]. Scientific research has proven that some tepal metabolites show potential antioxidant activity [3], antifungal [7], and antibacterial [8]. Tepals can be used as active ingredients in food supplements [9], cosmetic formulations [10], and as a potential natural color resource of anthocyanins for food [8]. Potential benefits include improving the sustainability of saffron production and the profitability of this industrial sector, taking advantage of high-value untapped biomass. This untapped biomass can generate benefits such as improving the sustainability and profitability of saffron production, strengthening the competitiveness and growth of the saffron agro-industry, and creating employment opportunities thanks to new recycling companies. Among the industries that use the waste of certain products, we find the biostimulant sector. By definition, biostimulants are substances and microorganisms discovered to regulate plant growth in several ways [11]. Research has shown their ability to enhance plant growth, nutrient efficiency, abiotic stress tolerance in plants, and crop quality traits [12], and other studies aim to identify and formulate products as alternatives to the use of chemicals [13–17]. Studies have shown their influence as biofertilizers [18–20]. Faced with the constraints of the chemical's use, which poses a risk to health and the environment, the principal objective was to develop new, economically, socially, and environmentally friendly agricultural techniques against biotic and abiotic stresses drought, salinity, pests, diseases, and weed infestations.

However, the bioactive molecules of tepals are supposed to have an effect biostimulant in recipient plants by spraying use. Therefore, the current study was planned to confirm the use of tepal extract as a biostimulant based on the morphological, physiological, and biochemical parameters of eggplant seedling. The study will be useful for developing a new biostimulant product and producing saffron by-products to expand the use of *C. sativus* L.

## 2. Materials and Methods

### 2.1. Tepal Production and Biochemical Analysis

#### 2.1.1. Experimental Condition and Production Collection

The experiments were conducted on *C. sativus* L. grown under three different sites (S) (S1:34° 52′ 30^″^ N, 2° 08′ 22^″^ W, 659 m; S2: 34° 52′ 04^″^ N, 2° 07′ 17^″^ W, 863 m; S3:34° 50′ 32^″^ N, 2° 10′ 23^″^ W, 1230 m) during 2018 and 2019 in the Oujda-Ahfir region of Morroco. The altitude was taken as the main factor in choosing the planting site. Saffron mother corms (5–7 g weight) were planted manually in 10–15 cm depth at 50 corms m^−2^ density on April 18, 2018, in each main plot (300 m^2^, 20 cm apart rows and 10 cm within rows). Irrigation management of saffron consists of six applications: in the middle of summer, the second in early October, the third in November, the fourth in late December, the fifth in early March, and the sixth in early April. After 180 days from the sowing date (October), tepals were separated by hand shortly after collecting in the field and were dried, in the dark, for 8–10 days, by the traditional methods at room temperature to avoid the degradation of the main bioactive molecules [21]. After drying, the tepals were crushed and stored at -20°C until use—the same harvesting and drying method was repeated in the same site's 2019 harvest plots. Stigmas from Taliouine (Morocco) were purchased, milled, and conserved at -20°C until each use.

#### 2.1.2. Biochemical Characteristics of Stigmas and Tepals

The tepal production from the three sites (S1, S2, and S3) of the two years (2018 and 2019) was mixed manually to have a sufficient quantity for the eggplant treatments.

The anthocyanin contents of tepals were measured according to the protocol explained by Ganjewala et al. [22]. The absorbance (Abs) of the samples was measured at 530 nm, and the results were expressed in microgram equivalent cyanidin 3-glucoside by one milliliter of extract (*μ*g CGE/mL).

Flavonol's contents were determined by measuring the absorbance at 360 nm, and the results were expressed in nanogram equivalent quercetin 3-glucoside by one milliliter of extract (*η*g QGE/mL).

The phenol concentration of tepals was determined using the Folin–Ciocalteu method [23]. The total polyphenol concentrations were expressed in a gallic acid microgram equivalent by one milliliter of extract (*μ*g GAE/mL).

The content of crocin, picrocrocin, and safranal of saffron stigmas was determined following ISO 3632 [24], and the results were expressed in microgram by one milliliter of extract.

### 2.2. Growth Conditions, Plant Material, and Experimental Design

#### 2.2.1. Eggplant Materials and Treatments

An experiment was conducted in the 2020 growing season in the polyethylene greenhouse of private property (34° 58′ 09^″^ N, 2° 06′ 47^″^ W). The mean temperature during sample collection was 27.3°C, and the maximum and minimum temperatures were approximately 39°C and 20°C. The plants were protected from excessive heat by shading and ventilation. Eggplant cultivar (BELLO F1) was sown in plastic trays until germination on April 15, 2020, at a temperature of (27°C ± 2), 70% relative humidity, and photoperiod of 16 h/8 h light/dark. After 40 days, the seedlings were transferred to plastic pots (33 × 18 cm) containing 65% sand, 30% peat (Floragard 50/50), and 5% gravel. The containers are placed on 90 cm apart rows and 70 cm within rows. There was no fertilizer application, and all the plants received the same volume of irrigation water. Ten days later, treatments were applied. ATE with three concentrations (TC1 = 1 mg/mL, TC2 = 2 mg/mL, and TC3 = 3 mg/mL) and a fourth treatment containing 2 mg/mL of tepal and 0.6 mg/mL of saffron stigma (T+S) were applied both as a foliar spray. The choice of concentration was based on the tests carried out before, under the same conditions, on tomato seedling. Distilled water is taken as a control treatment. Each replicate set contained three seedlings, and the experiment had three replications realized in a completely randomized experimental design. Each plant received a 100 mL volume of the extract of each concentration. The procedure was repeated three times every 15 days, keeping the same concentration and the same volume as the first treatment. One week after the third treatment, when the biostimulant effect was visually observed, leaf samples receiving the three spray treatments were collected to determine the biochemical and physiological indices and the antioxidant activity.

#### 2.2.2. Preparation of the Aqueous Extracts

Before each foliar spraying, the concentrations of tepal powder used for the study were left 24 h in a volume of 1 L of distilled water for each level: 1 mg/mL, 2 mg/mL, and 3 mg/mL. The extraction was done in the dark. The mixture powder of 2 g of tepals and 0.6 g of stigmas was extracted in one liter of distilled water for 24 hours in the dark.

### 2.3. Morphological, Biochemical, and Physiological Indices of Eggplants

#### 2.3.1. Plant Height

Plant height was recorded in centimeters using a measuring tape. The measurements of plant height were made one week after the last spray treatment.

#### 2.3.2. Chlorophyll Contents

The chlorophyll content was determined by crushing 0.2 g fresh leaf sample in 20 mL of 80% acetone and placed at room temperature for 48 h in the dark. A spectrophotometer was used to observe the absorbance at 663, 645, and 652 nm of the samples [25]. Chlorophyll a, b, and total chlorophyll were calculated using the following formula:

(1) $$\begin{array}{c} \text{Chlorophyll a }\left(\text{Chl a}\right)=\left(12.7\times \text{Abs}663\right)-\left(2.69\times \text{Abs}645\right),\text{Chlorophyll b }\left(\text{Chl b}\right)=\left(22.99\times \text{Abs}645\right)-\left(4,68\times \text{Abs}663\right)\text{Total chlorophyll}=\left(\text{Chla}+\text{Chlb}\right). \end{array}$$

#### 2.3.3. Lipid Peroxidation (MDA)

The MDA was quantified using the method by Heath and Packer [26]. About 3 g of tissue was homogenized in 5 mL of 5% (w/v) trichloroacetic acid, and the homogenate was centrifuged for 15 min at room temperature. The supernatant was mixed with an equal volume of thiobarbituric acid (0.5% in 20% (w/v) trichloroacetic acid). The mixture was heated for 25 min at 95°C, followed by centrifugation for 5 min at 7500 × g to clarify the solution. The absorbance at the wavelength of 532 nm was measured and subtracted from the absorbance at 600 nm. The MDA concentration was calculated using the extinction coefficient of 155/mM cm and expressed in nanomoles per gram fresh weight (FW).

#### 2.3.4. L-Ascorbic Acid Content

The L-ascorbic acid content estimation was carried out using the method described by Mau et al. [27] with some modification. Fresh leaves (12.5 g) were homogenized in 50 mL of oxalic acid (1%) followed by stirring for 15 min, and filtration—3 mL—of the filtrate was mixed in 1 mL of 2.6-dichlorophenolindophenol (DCPIP) 5 mM. The absorbance was then measured at 515 nm after 15 s. The L-ascorbic acid concentrations were expressed in milligrams per gram FW of ascorbic acid equivalent (AscAE).

#### 2.3.5. Total Polyphenols, Soluble Sugars, and In-Vitro Antioxidant Activity

*(1) Hydromethanolic Extract Preparation*. Each fresh leaf samples were extracted with methanol (80%, v/v) and macerated for 48 h with an agitator maintained at room temperature. The extract was filtered, and concentrations of 0.1 g/mL were prepared with methanol (80%, v/v) and stored in a brown bottle at −20°C until use.

*(2) Total Polyphenols*. The total polyphenol concentrations were determined using the Folin–Ciocalteu method [23]. Dilute extract (500 *μ*L) from each sample was homogenized in 3.75 mL of distilled water and 250 *μ*L of Folin–Ciocalteu reagent. After 5 min, 0.5 mL of sodium carbonate solution (20%) was added, and the mixture was incubated in the dark for 30 min. The absorbance was measured at 760 nm. The total polyphenol concentrations were expressed in milligrams per 100 g FW of gallic acid equivalent (GAE).

*(3) Soluble Sugars*. Total soluble sugars were determined using the absorbance of the sample extracts against glucose and anthrone dissolved in H_2_SO_4_ following the method described by Ali et al. [28]. The absorbance was measured at 620 nm, and the results were expressed in milligrams per gram of FW.

*(4) The DPPH Radical Scavenging Activity*. The DPPH radical scavenging activity was established using the method suggested by Braca et al. [29] with some modification. About 0.1 mL of the extract (*C* = 0.05 g/mL) was added to 2.9 mL of DPPH (6.10^−5^) diluted in methanol, and the absorbance was measured at 517 nm after incubation for 30 min at room temperature. Distilled water was used as the control. The percent inhibition activity was calculated according to the equation:

(2) $$\begin{array}{c} \left(\%\right)\text{ Inhibition activity }\left(\text{DPPH}\right)=\left(\left(\text{Abs control}-\text{Abs extract}\right)/\left(\text{Abs control}\right)\right)\times 100. \end{array}$$

*(5) β-Carotene Bleaching Test*. Antioxidant activity was determined using the *β*-carotene bleaching method described by Marco [30] with some modifications. About 2 *μ*L of linoleic acid, 100 *μ*L of 10% Tween 20, 2.8 mL of distilled water, and 30 *μ*L of a *β*-carotene solution was added to the samples (0.1 mL; *C* = 0.05 g/mL). The mixtures are vigorously mixed by vortexing, and their initial absorbance (Ai) was measured at 470 nm. After incubation at 50°C for 2 h, the absorbance was measured (Af), and the percentage of oxidized *β*-carotene was calculated as:

(3) $$\begin{array}{c} \left(\%\right)\text{ oxidized }\beta -\text{carotene}=\left(\text{Ai}-\text{Af}/\text{Ai}\right)\times 100. \end{array}$$

*(6) Determination of Ferric Reducing Power Assay*. The FRAP (ferric reducing power activity) was performed according to the method described by Dehpour et al. [31]. Briefly, about 1.25 mL of phosphate buffer (0.2 M, pH 6.6) and 1.25 mL of potassium ferricyanide was added to 0.5 mL of the samples. After incubation at 50°C for 20 min, 1.25 mL of trichloroacetic acid (10% w/v) was added. Then, about 1.25 mL of the supernatant solution was homogenized, a solution containing 1.25 mL of distilled water and 0.25 mL of ferric chloride (0.1% w/v). The absorbance was measured at 700 nm. The results expressed were expressed in milligrams per 100 g FW of Trolox equivalent (TROE).

*(7) Total Antioxidant (Phosphomolybdate Assay)*. The total antioxidant activity was estimated based on the phosphomolybdate reagent and was measured using the method described by Umamaheswari and Chatterjee [32]. About 100 *μ*L of the samples was homogenized in 1 mL of the phosphomolybdate reagent. The solution was incubated in a water bath at 95°C for 90 min, and the absorbance was measured at 695 nm. The results are expressed in milligrams equivalent (Trolox and *α*-tocopherol) by 100 grams of fresh matter (mg TROE; TOCE/100 g FW).

### 2.4. Statistical Analysis

All figures and statistical analyses were performed using the SPSS Statistics 17.0 software. The data were expressed as the mean ± standard deviation (SD) of triplicate independent experiments and analyzed using a one-way analysis of variance (ANOVA). *p* ≤ 0.05 was treated to be statistically significant—the Student-Newman-Keuls (SNK) test was used to classify averages using letters with substantial differences. The relation between the analyzed parameters was realized using a Pearson correlation.

## 3. Results

### 3.1. Biochemical Content of Extracts Used for Treatment

Table 1 presents the main components of tepals and saffron stigma extracts of each concentration used for foliar treatments. The T+S treatment contains the highest concentration of polyphenol; however, the TC3 treatment has the highest concentration of anthocyanins and flavonols.

### 3.2. Plant Height

**Figure 1:**
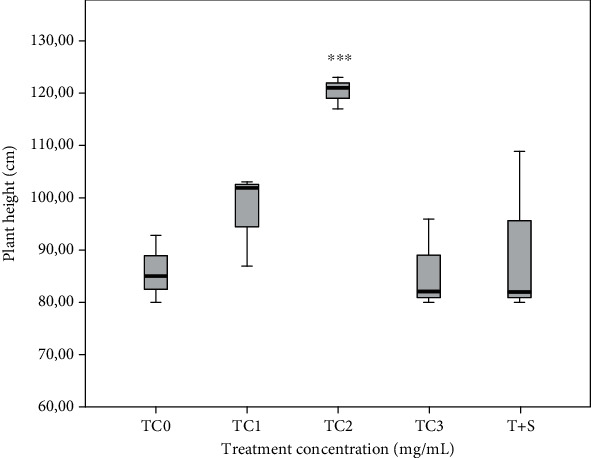
Tepal extracts affect the eggplant seedling height. The measurements were made one week after the last spray treatment. The data were recorded for a total of three plants by treatment replicated three times. The tepal concentrations (TC) was represented as TC0 = 0 (control), TC1 = 1 mg/mL, TC2 = 2 mg/mL, and TC3 = 3 mg/mL. T + S = 2 mg/mL of tepal extract + 0.6 mg/mL of saffron stigmas. The boxplot represents averages and error lines of one standard deviation.^∗∗∗^ indicates the significant differences at *p* ≤ 0.01 based on the SNK test.

**Figure 2:**
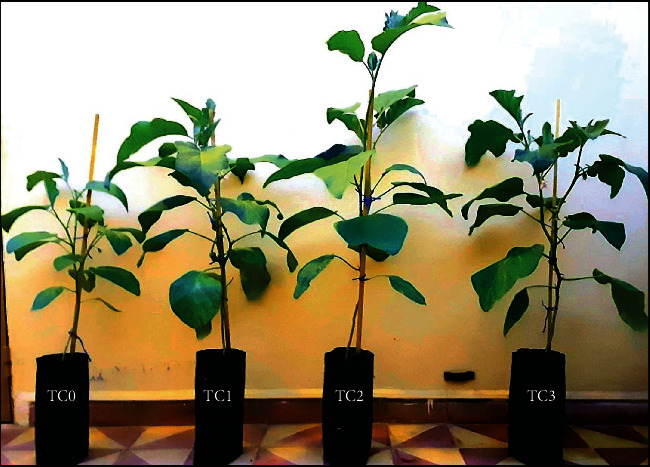
The aqueous tepal extract application influences the plant height of eggplant seedling. The pictures were taken one week after the last spray treatment. The tepal concentrations (TC) were represented as TC0 = 0 (control), TC1 = 1 mg/mL, TC2 = 2 mg/mL, and TC3 = 3 mg/mL.

The plant height is insignificantly different (*p* ≤ 0.05) in the TC1, TC3, T+S plant groups. However, a significant increase (*p* ≤ 0.05) in plant height was recorded in the TC2 plant group compared to the control group (Figure 1; Figure 2).

### 3.3. Biochemical and Physiological Indices and Antioxidant Activities

**Figure 3:**
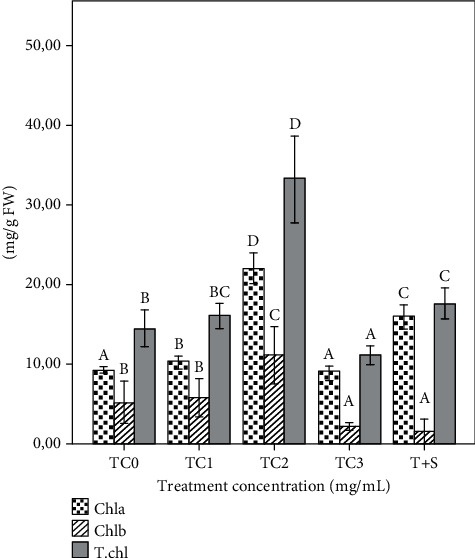
The chlorophyll content of eggplant affected by foliar spray of tepal extract and tepal with saffron stigma extract (T+S). Bars represent means and standard errors of triplicates. Means followed by the same letters are not significantly different from each other at *p* ≤ 0.05 based on the SNK test. The tepal concentrations (TC) was represented as TC0 = 0 (control), TC1 = 1 mg/mL, TC2 = 2 mg/mL, and TC3 = 3 mg/mL. T + S = mg/mL of tepal extract + 0.6 mg/mL of saffron stigmas. Chla: chlorophyll a; Chlb: chlorophyll b; T.chl: total chlorophyll; FW: fresh weight.

The total chlorophyll content was significantly different in the TC1, TC2, TC3, and T+S plant groups. However, a significant (*p* ≤ 0.05) reduction in the total chlorophyll and Chl b content was recorded in the TC3 plant groups compared to the control, while the highest Chl a and Chl b content was observed (*p* ≤ 0.05) in the TC2 plant group compared to the control (Figure 3).

**Figure 4:**
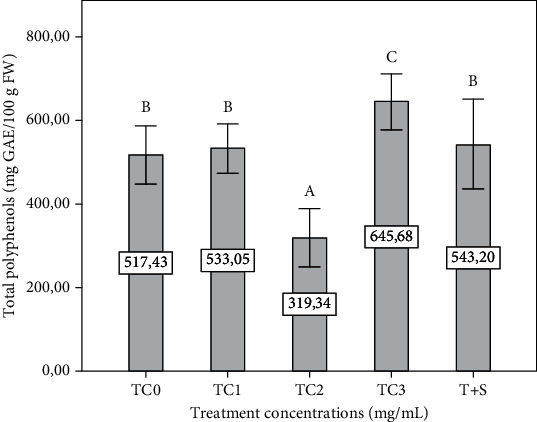
Effect of different extract concentrations on total polyphenols of eggplant leaves. The tepal concentrations (TC) were represented as TC0 = 0 (control), TC1 = 1 mg/mL, TC2 = 2 mg/mL, and TC3 = 3 mg/mL. T + S = 2 mg/mL of tepal extract + 0.6 mg/mL of saffron stigmas. The data are the means of three replicates. Vertical bars represent the standard deviation of the mean. Values not sharing a common letter indicate a significant difference at *p* ≤ 0.05, based on the SNK test. GAE: gallic acid equivalent; FW: fresh weight.

Total phenolics were significantly (*p* ≤ 0.05) increased in the TC3 plant group and decreased in the TC2 plant group compared to the control. However, the total phenolics were insignificantly (*p* ≤ 0.05) decreased in the TC1 and T+S plant groups compared to the control (Figure 4).

**Figure 5:**
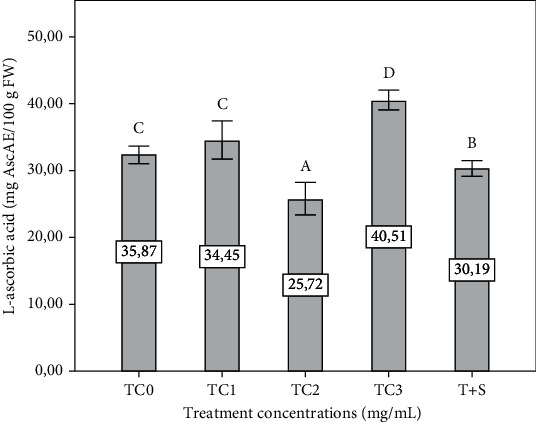
Effect of extract concentrations on the L-ascorbic acid content of eggplant seedling. The tepal concentrations (TC) were represented as TC0 = 0 (control), TC1 = 1 mg/mL, TC2 = 2 mg/mL, and TC3 = 3 mg/mL. T + S = 2 mg/mL of tepal extract + 0.6 mg/mL of saffron stigmas. Vertical bars represent the standard deviation of the mean. Values not sharing a common letter indicate a significant difference at *p* ≤ 0.05, based on the SNK test. AscAE: ascorbic acid equivalent, FW: fresh weight.

L-ascorbic acid content was significantly increased in the TC3 plant group and decreased in the TC2 and T+S plant groups compared to the control (*p* ≤ 0.05). However, L-ascorbic acid was insignificantly (*p* ≤ 0.05) fell in the TC1 and T+S plant groups compared to the control (Figure 5).

**Figure 6:**
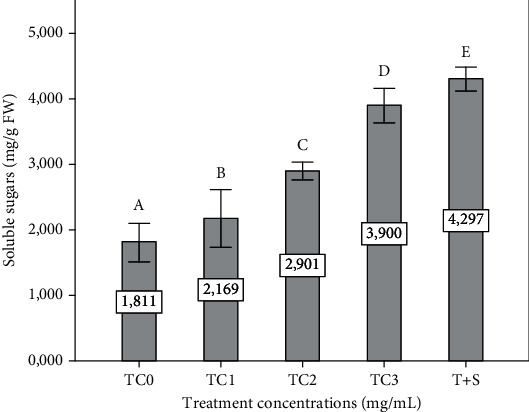
The soluble sugar content of eggplant affected by foliar spray of extracts. The tepal concentrations (TC) were represented as TC0 = 0 (control), TC1 = 1 mg/mL, TC2 = 2 mg/mL, and TC3 = 3 mg/mL. T + S = 2 mg/mL of tepal extract + 0.6 mg/mL of saffron stigmas. Vertical bars represent the standard deviation of the mean. Values not sharing a common letter indicate a significant difference at *p* ≤ 0.05, based on the SNK test. FW: fresh weight.

The soluble sugar content was significantly decreased in the TC1, TC2, TC3, and T+S plant groups compared to the control (*p* ≤ 0.05). Interestingly, the effect of ATE application in soluble sugar content was linear to the increasing concentration (Figure 6).

**Figure 7:**
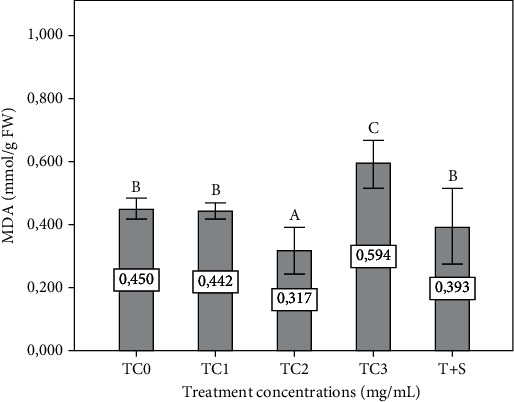
Effect of extracts on lipid peroxidation (MDA) of eggplant seedling by foliar spray. The tepal concentrations (TC) were represented as TC0 = 0 (control), TC1 = 1 mg/mL, TC2 = 2 mg/mL, and TC3 = 3 mg/mL. T + S = 2 mg/mL of tepal extract + 0.6 mg/mL of saffron stigmas. The data are the mean ± standard errors of three replicates. The column followed by different letters shows a significant difference at *p* ≤ 0.05 significance level between treatments according to the SNK test. FW: fresh weight; MDA: malondialdehyde.

The MDA content was significantly increased in the TC3 plant group and decreased in the TC2 plant group compared to the control (*p* ≤ 0.05). However, MDA was insignificantly (*p* ≤ 0.05) decreased in the TC1 and T+S plant groups compared to the control (Figure 7).

**Figure 8:**
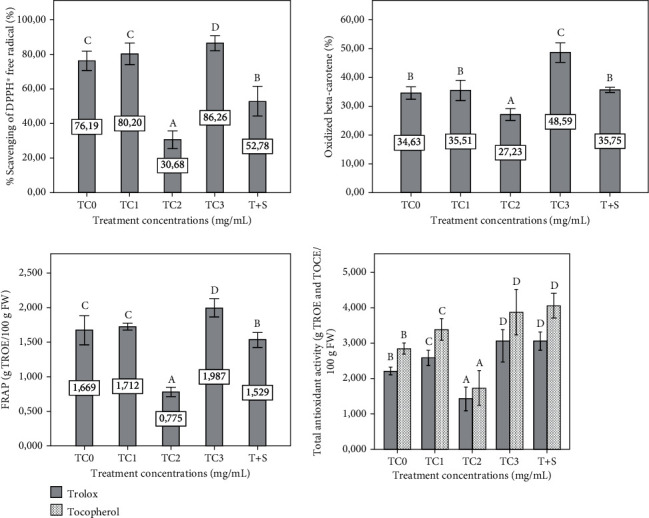
Effect of extract concentrations on antioxidant activity (DPPH, oxidized *β*-carotene, FRAP, and total antioxidant) of the eggplant seedling. The tepal concentrations (TC) were represented as TC0 = 0 (control), TC1 = 1 mg/mL, TC2 = 2 mg/mL, and TC3 = 3 mg/mL. T + S = 2 mg/mL of tepal extract + 0.6 mg/mL of saffron stigmas. The data are the means of the three replicates. Vertical bars represent the standard deviation of the mean. Values not sharing a common letter indicate a significant difference at *p* ≤ 0.05 based on the SNK test. FW: fresh weight; DPPH: 1,1-diphenyl-2-picrylhydrazyl; TROE: Trolox equivalent; TOCE: *α*-tocopherol equivalent; FRAP: ferric reducing antioxidant power.

The antioxidant properties of all four activities related (DPPH, *β*-carotene, FRAP, and total antioxidant activities) were significantly decreased in the TC2 and T+S plant groups compared to the control (*p* ≤ 0.05) except for the total antioxidant activities; the properties were increased in the T+S groups. However, the antioxidant properties were significantly increased in the TC3 plant group compared to the control (*p* ≤ 0.05), while the insignificant difference was found in the antioxidant activities in the TC1 group (*p* ≤ 0.05), except for the total antioxidant activities, compared to the control (Figure 8).

## 4. Discussion

The present study's objective was to valorize the *C. sativus* L. tepals and take advantage of its bioactive components to use them as a biostimulant alternative to chemical products that pose a risk to the environment and humanity. Therefore, three concentrations of tepal extract (1 mg/mL, 2 mg/mL, and 3 mg/mL) are chosen to evaluate the ATE effect in some morphological, physiological, and biochemical parameters of the eggplant seedling under greenhouse conditions in a completely randomized block with three repetitions for each group which contains three plants, after three treatments every 15 days.

The foliar ATE application influenced the growth parameter of the eggplant plants. Plant seedlings reacted to different concentrations of ATE, perhaps by the fact that ATE contains various growth-promoting compounds such as amino acids, fibers, and essential minerals (Ca, K, Mg, P, and Fe), in particular in the iron [33] required for plant growth [34, 35]. Tepals are also rich sources of phenolic and bioactive compounds such as flavonol and anthocyanins [36, 37]. The improvement in the plant height in this study suggests that the extract of tepal may act as a plant growth promoter at TC2 = 2 mg/mL after three applications, which contains 8.05 *μ*g CGE/mL anthocyanin, 13.26 *μ*g GAE/mL polyphenol, and 4.26 *η*g/mL flavonol. ATE and T+S contain secondary metabolites, especially polyphenol, which is a potent antioxidant, and at higher concentrations, it may inhibit the growth of the recipient plants. A higher level of these antioxidant compounds tends to disrupt the recipient plants' physiological balance, leading to the respective growth inhibition [38]. The higher concentration atTC3 = 3 mg/mLcontains 19.89 *μ*g/mL polyphenol, and the spray application of T+S has 27.62 *μ*g/mL polyphenol which hampered the plant growth after three foliar applications.

The ATE treatment also influenced the photosynthetic pigments of the plants. Photosynthesis is one of the main processes of plant metabolism, strongly influenced by different external signs. It is one of the critical elements of photosynthesis processes necessary to absorb sunlight [39]. It occurs in chloroplasts in all photosynthetic plant tissues, Chl a, and Chl b bind to light-collecting complex (LHC) proteins via weak noncovalent bonds. The light energy captured by the LHC protein is transferred to the thylakoid chloroplast membrane [40]. The ATE application has a positive effect on chlorophyll content: the TC2 concentration = 2 mg/mL significantly improves the chlorophyll content compared to the control. This increase in pigment content may result from reduced chlorophyll degradation, which may be related mainly to polyphenol in the aqueous extract, and many other molecules that can increase the pigment content [33]. They can also activate enzymes responsible for the regulation and photosynthetic reduction of carbon and the chloroplast protection against oxidative damage. Also, they can include compounds that play a photoprotective role by scavenging reactive oxygen species. The exogenous application of biostimulant has been shown to activate specific genes involved in the transcription of proteins for photosynthetic processes [41]. However, the foliar ATE application at 3 mg/mL results in a significant reduction in total chlorophyll. This finding indicates that ATE at high levels adversely affects photosynthetic pigments by destroying Chl a and Chl b. Chlorophyll reduction may result from the degradation of LHC proteins [42] and disruption of envelope degradation of thylakoids and chloroplasts [43]. However, there was a positive correlation (Table 2) between the plant height and chlorophyll content (*r* = 0.827^∗∗^). Therefore, we can conclude that the foliar application of ATE accelerates plant growth by stimulating the production of photosynthetic pigments.

On the other hand, the leaves' ascorbic acid content was influenced after the ATE application. Ascorbic acid is a water-soluble antioxidant with exceptional importance in plant cells, protecting plants from oxidative damage [44], and the leaves are the primary source of ascorbic acid in plants [45].

The reducing properties of ascorbic acid come from the enediol group, thanks to which it can directly remove various forms of ROS (reactive oxygen species). However, as a substrate of ascorbate peroxidase, ascorbic acid contributes to protecting plant cells from oxidative stress and eliminating ROS [46]. Besides, the increase in ascorbic acid synthesis in terms of oxidative stress is associated with its role in the ascorbate-glutathione cycle [47]. According to our results, the highest ascorbic acid content observed in plants treated with TC3 = 3 mg/mL indicates their more increased need for antioxidant defense. On the other hand, T+S caused a slight decrease of L-ascorbic acid content in the leaves, and TC = 2 mg/mL caused a sharp reduction in the concentration of ascorbic acid compared to the control. We can hypothesize that the sugars present in biostimulants improve the biosynthesis of low molecular weight antioxidant compounds such as L-ascorbic acid [48]; this is the case of our extract, which contains an intense concentration of sugar, especially glucose. Our results indicate that 2 mg/mL ATE and T+S considerably reduce oxidative stress, resulting in a lower ascorbate concentration.

The application of ATE affected the MDA, antioxidant activity of the eggplant plants, and polyphenol concentrations. Oxidative stress, which is generally caused by the imbalance in the accumulation and elimination of ROS molecules, was measured by evaluating the level of lipid peroxidation and antioxidant properties in the plants' leaves. Lipid peroxidation, an essential symptom of oxidative stress in plants [49], was measured as the MDA content. Foliar application of tepal extract at 3 mg/mL resulted in a significant MDA content accumulation. These physiological alterations provide a basis for understanding the effect of polyphenol and the other molecules present in the extract. Excessive accumulation of ROS leads to oxidative damage, which results in lipid peroxidation and membrane leakage at the cellular level in plants [50]. It has often been reported that the active state of antioxidants is a sign of stress response in plants, explained in our study by the negative correlation (Table 2) between MDA and plant height (*r* = −0.666^∗∗^). However, a significant decrease in the MDA content was observed in the plants treated with 2 mg/mL compared to the control treatment: during plant growth, a balance of prooxidants and antioxidants was found in a continuum, and any kind of imbalance can result in oxidative stress causing devastation at the cellular level [50, 51]. Besides, ROS are typical by-products of plants that undergo natural cellular changes. An increased ROS production may result from external signs that the plant may receive, considering that our foliar treatments are part of the outward symptoms. The modification of the insignificant lipid peroxidation allows us to ensure a state of immune or induced defense in the eggplant plants. ATE is a rich source of phenolic compounds. It can be suggested these phenolic compounds are potent antioxidants and biologically active, proposing to have altered the physiology of eggplant plants, causing an imbalance of redox activity at the cellular level. In addition to lipid peroxidation, the study of antioxidant activities is often considered to be indications of severe stress or stress-type reactions which explains in our study the positive correlation (Table 2) between MDA and DPPH activity (*r* = 0.859^∗∗^), *β*-carotene (*r* = 0.931^∗∗^), and FRAP (*r* = 0.844^∗∗^). Free radicals are known to play a role in a wide variety of stressful situations. Antioxidants fight against free radicals to protect the plant. They exert their action either by trapping reactive oxygen species or protecting antioxidant defense mechanisms [32]. Our research found that 2 mg/mL ATE decreased the polyphenols' content, and the antioxidant activity was measured with the DPPH, FRAP, *β*-carotene, and phosphomolybdate assay. It was shown that the activity of DPPH correlated with the content of polyphenols, and the *β*-carotene activity correlated with ascorbic acid. At the same time, total antioxidant activity is positively related to tannin concentrations [52]. The phenolic's potential benefit is mainly attributed to its antioxidant activity by donating a hydrogen atom of the aromatic hydroxyl group to free radicals. Therefore, it can be assumed that ATE 2 mg/mL strengthened the plants: the plants do not need to produce polyphenols. Polyphenols are not involved in the growth and development of plants [53], and this is confirmed in our study, given the negative correlation (*r* = −0.743^∗∗^) between the polyphenol content and the plant height (Table 2). However, phenolic acids are directly involved in the response of plants to different types of stress. The improved metabolism of phenylpropanoids and the number of phenolic compounds can be affected by various environmental factors; the synthesis of isoflavones and several flavonoids are generated when plants are infected or damaged, as well as under low-stress conditions [54–56] as in the case of high concentration TC = 3 mg/mL which has shown stress on the plant; based on the analysis of MDA and secondary metabolites, in particular ascorbic acid, and according to the data, the increased content of ascorbic acid is closely associated with the concentration of phenol due to the stimulation of the pathway phenylpropanoid, due to which the antioxidant capacity also increases [57] confirmed in our study by a positive correlation between polyphenols and ascorbic acid (Table 2). The phenolic content enhanced the DPPH antioxidant activity, concluding that these phenolics in the plants' leaves were antioxidant. However, the increase in polyphenol could be due to phenolic polymerization, phenolic glycosylation, or phenolic oxidation [58–60]. Other studies have explained that the increase in antioxidant activity is due to increased solubilized ferulic acid esters and an increased release of phenolic compounds [61]. So we can conclude, following the positive correlations between the antioxidant properties of plants and stress indices (Table 2) such as MDA and ascorbic acid, that the antioxidant activity was a marker to confirm the stress state of the plant as well as an essential parameter to understand the plant's response to biostimulant products.

## 5. Conclusion

The results of this study demonstrate that ATE alters the antioxidant mechanisms and enhances the growth in eggplants. ATE applied at 2 mg/mL can be used in specialized horticultural practices as a biostimulant for organic farming and farmers' improved income for practicing saffron. The preparation has almost no hazardous side effects and offers ecofriendly. Additional studies are needed to evaluate ATE's most essential bioactive constituents as flavonoids and anthocyanins to identify the potent polyphenol responsible for biostimulation.

## Figures and Tables

**Table 1:** Content by each concentration of the main bioactive molecules of the extracts used for foliar treatment. Data are mean ± standard deviation. TC1 = 1 mg/mL, TC2 = 2 mg/mL, and TC3 = 3 mg/mL. T + S = 2 mg/mL of tepal extract + 0.6 mg/mL of saffron stigmas. CGE: cyanidin 3-glucoside equivalent; QGE: quercetin 3-glucoside equivalent; GAE: gallic acid equivalent.

Concentration (mg/mL)	Phenol (*μ*g GAE/mL)	Anthocyanin (*μ*g CGE/mL)	Flavonol (*η*g QGE/mL)	Crocin(*μ*g/mL)	Picrocrocin(*μ*g/mL)	Safranal(*μ*g/mL)
TC1	6.63 ± 0.14	4.02 ± 1.02	2.13 ± 0.68	-	-	-
TC2	13.26 ± 0.28	8.05 ± 2.04	4.26 ± 1.36	-	-	-
TC3	19.89 ± 0.56	12.07 ± 4.08	6.39 ± 2.72	-	-	-
T+S	27.62 ± 2.15	8.05 ± 2.04	5.06 ± 2.07	184.56 ± 1.82	4.89 ± 0.18	62.43 ± 0.35

**Table 2:** Correlation between the analyzed biochemical and physiological indices of eggplant leaves. DPPH: 1,1-diphenyl-2-picrylhydrazyl; MDA: malondialdehyde; AAsc: acid ascorbic, FRAP: ferric reducing antioxidant power; Chl: chlorophyll; T.Chl: total chlorophyll; TotTrolox: total antioxidant in Trolox equivalent; TotToc: total antioxidant in *α*-tocopherol equivalent; Sugars: soluble sugars.

	MDA	AAsc	Polyphenol	DPPH	*β*-carotene	FRAP	Chla	Chlb	T.chl	Height	Sugars	TotTrolox	TotTocopherol
MDA	1												
AAsc	.917^∗∗^	1											
Polyphenol	.818^∗∗^	.856^∗∗^	1										
DPPH	.859^∗∗^	.940^∗∗^	.832^∗∗^	1									
*β*-Carotene	.931^∗∗^	.874^∗∗^	.874^∗∗^	.759^∗∗^	1								
FRAP	.844^∗∗^	.921^∗∗^	.947^∗∗^	.936^∗∗^	.854^∗∗^	1							
Chla	-.811^∗∗^	-.932^∗∗^	-.819^∗∗^	-.980^∗∗^	-.708^∗∗^	-.931^∗∗^	1						
Chlb	-.615^∗^	-.642^∗∗^	-.888^∗∗^	-.590^∗^	-.744^∗∗^	-.811^∗∗^	.611^∗^	1					
T.chl	-.812^∗∗^	-.903^∗∗^	-.940^∗∗^	-.911^∗∗^	-.802^∗∗^	-.979^∗∗^	.934^∗∗^	.854^∗∗^	1				
Height	-.666^∗∗^	-.683^∗∗^	-.743^∗∗^	-.712^∗∗^	-.607^∗^	-.765^∗∗^	.742^∗∗^	.749^∗∗^	.827^∗∗^	1			
Sugars	.172	-.031	.290	-.192	.423	.085	.248	-.503	-.065	-.105	1		
TotTrolox	.571^∗^	.593^∗^	.880^∗∗^	.602^∗^	.727^∗∗^	.815^∗∗^	-.586^∗^	-.935^∗∗^	-.808^∗∗^	-.693^∗∗^	.516^∗^	1	
TotToc	.571^∗^	.593^∗^	.880^∗∗^	.602^∗^	.727^∗∗^	.815^∗∗^	-.586^∗^	-.935^∗∗^	-.808^∗∗^	-.693^∗∗^	.516^∗^	1.000^∗∗^	1

^∗∗^Correlation is significant at the 0.01 level (2-tailed).^∗^Correlation is significant at the 0.05 level (2-tailed).

## Data Availability

The data used to support the findings of this study have been deposited in 10.1016/j.aoas.2019.10.002
